# Supplementary material for: Are carbon emissions trading and green financial instruments synergistic? -Comprehensive quantitative research based on content analysis
Source: PLoS One. 2024 Mar 7;19(3):e0298601. doi: 10.1371/journal.pone.0298601 (PMC10919601; doi:10.1371/journal.pone.0298601)
Supplement: S1 Data — (ZIP) [file pone.0298601.s001.zip › Data/地级市Z_F工作报告.zip!/║╙▒▒/╟╪╗╩╡║╩╨/└┤╫╘╣1⁄2╓┌║┼í╛┬φ┐╦╩2╛▌═°í┐_2016.docx]

各位代表：

现在，我代表市人民政府向大会作工作报告，请各位代表连同《秦皇岛市国民经济和社会发展第十三个五年规划纲要(草案)》一并审议，并请市政协委员和列席会议的同志提出意见。

一、“十二五”时期经济社会发展回顾

2011年以来的五年，是我市积极应对严峻复杂形势、经济持续平稳健康发展的五年，是社会事业全面进步、人民生活不断改善的五年。特别是党的十八大以来，在省委、省政府和市委坚强领导下，全市上下认真贯彻落实习近平总书记系列重要讲话精神，坚持稳中求进工作总基调，主动适应经济发展新常态，攻坚克难、奋力前行，在全面建成小康社会进程中迈出新步伐。

——经济实力稳步增长。坚持把握平衡点，培育增长点，着力扩内需、稳增长、提质量，促进经济稳健运行。2015年实现生产总值1250.44亿元，按可比价格计算，比上年增长5.5%，比2010年增长44.8%;人均生产总值突破4万元，比2010年增长40.8%;一般公共预算收入114.36亿元，按可比口径比上年增长6.5%，比2010年增长58.8%，占生产总值的比重由2010年的7.74%提高到9.15%。2015年实现社会消费品零售总额631.33亿元，比上年增长9.3%，比2010年增长88.5%;完成全社会固定资产投资892.45亿元，比上年增长10.4%，比2010年增长76.5%，中信戴卡产业园、中铁山桥产业园等重大项目建成投产;城乡居民人均可支配收入分别达到28158元和10782元，分别比上年增长8.1%和8.2%，比2010年增长65.4%和80.2%，均高于经济增幅;全市常住人口城镇化率达到54.07%。省设区市全面建成小康社会进程监测评价结果，2014年我市小康社会综合指数居全省第二位。政府工作报告

——产业结构进一步优化。坚持抓重点、攻难点，大力化解过剩产能、调整产业结构，推进发展方式加快转变。累计压减炼铁产能375万吨、炼钢产能210万吨、水泥产能494万吨，预计单位生产总值能耗累计下降20.9%;规模以上工业增加值中，玻璃、钢铁、水泥等产业占比由2010年的31.1%降到23%，中高端产业占比由30.4%提高到44.9%，其中装备制造、电子信息制造业占比分别达到39.5%和5.4%;高新技术企业、科技小巨人企业、科技型中小企业分别达到93家、59家和1030家。努力壮大现代服务业，2015年完成服务业增加值627.72亿元，比2010年增长45.6%;实现旅游总收入362.4亿元，比2010年增长145.9%;港口货物吞吐量达到2.53亿吨，集装箱突破50万标箱;秦皇岛能源交易中心揭牌运营，国际粮食产业及秦皇岛大宗商品交易中心项目签约，秦皇岛港成为国家进境粮食指定口岸。积极发展现代农业，特色农业加快发展，现代农业园区建设全面推进，农业产业化经营率保持全省领先。

——协同发展初见成效。坚持立足优势、精准对接、务实融入，明确“一都三区一枢纽”战略定位，推动交通、产业、生态等重点领域率先突破，努力建设京津冀重要节点城市。京唐秦城际铁路、京秦高速二通道等一批重大基础设施工程启动实施，我市开通至武汉、太原、石家庄等地的高铁，真正迎来“高铁时代”。国家级北戴河生命健康产业创新示范区建设有望获批，北戴河生态建设及功能疏解提升工程获得国家大力支持，广济医院迁建、与北大未名集团合作项目动工，侨梦苑侨商产业聚集区正式揭牌。中关村海淀园秦皇岛分园入驻企业达到68家，中国环境科学研究院大气污染防治研究中心落户北戴河新区，天津肿瘤医院秦皇岛分院正式挂牌，与北京顺鑫集团、首创集团等合作进展顺利。完成区划调整，抚宁撤县设区，城市发展空间实现跨越拓展。

——改革开放深入推进。坚持除旧更新、攻坚克难，着力破除体制机制障碍。完成新一轮政府机构改革，全面清理非行政审批事项，市本级行政审批事项精简 62.1%，将38项与项目建设相关的管理权限下放到城市区，在秦皇岛开发区、北戴河新区设立行政审批局。中建材凯盛科技重组耀华集团进入国有股权划转程序。农村综合改革深入推进，农村集体土地所有权确权登记全部完成，农村土地承包经营权确权176.7万亩，占全部承包耕地的61.1%，在全省率先实现任务县区农村产权交易中心全覆盖。财税金融体制改革继续深化，“营改增”有序推进;市商业银行更名为秦皇岛银行，民生银行、河北银行、沧州银行、承德银行在我市设立分行;成立秦皇岛银行科技支行，设立中小微企业贷款风险补偿资金和红土创业基金，新增上市挂牌企业27家;争取棚改低息贷款79亿元、置换债券81.75亿元、新增债券8.62亿元。供销社综合改革、户籍制度改革等重点改革扎实推进，科技、教育、文化、卫生等领域改革迈出新步伐。对内对外开放不断拓展，累计引进外资36.4亿美元，完成外贸进出口总额220.5亿美元，其中出口129.8亿美元，均居全省前列。全面实施商事制度改革，每万人拥有市场主体523.8家，比2010年增加207.5家;预计民营经济实现增加值占地区生产总值的比重65%左右。

——统筹城乡发展步伐加快。坚持着眼长远打基础，以城带乡促协调，积极稳妥推进新型城镇化。津秦客专、承秦高速及连接线建成通车，北戴河火车站完成改造，北戴河机场具备通航条件，兴凯湖路“两桥一路”、民族路和北环路上跨铁路桥等重点工程竣工。完成国省干线大修工程50项、973公里，改造农村公路 1947公里。北戴河新区污水处理厂建成投用，引青三期工程、北戴河西部水厂、海港区北部、西部污水处理厂等项目加快建设。完成海港西部和北部综合片区、北戴河车站片区、山海关道南片区等城市重点片区改造建设，黄金海岸自然保护区规划调整获批，北戴河国家级风景名胜区通过复查验收，城市人居环境和功能品质明显提升。拓展县城空间、完善县城功能、提升县城形象，各县垃圾处理厂达到二级及以上标准，各县县城均成为省级园林县城，青龙祖山镇等8个建制镇被列为全国重点镇。积极推进农村面貌改造提升和美丽乡村建设，完成75个村新民居建设，创建15个省级美丽乡村、20个山区综合开发示范区。

——生态环境显著改善。坚持铁腕治污、重拳出击，以前所未有的决心和力度推进污染防治和生态建设。大力压能、减煤、控车、降尘、治企、增绿，推进钢铁、水泥、电力、玻璃行业污染治理，推广农村清洁能源开发利用，淘汰黄标车4.51万辆，更换新能源公交车484辆，拆除砖瓦窑77座，淘汰燃煤锅炉 598台，累计完成工程减排项目928个，热电厂实现超低排放。去年空气质量二级以上天数达到269天，比2013年增加60天，细颗粒物(PM2.5) 下降26.2%。基本完成北戴河近岸海域环境综合整治三年行动计划，大力治理16条入海河流，关停255家排污企业，整治养殖场183个，对城区112个排污口和54个污染源采取针对性工程措施，海水质量和入海河口水质持续向好，海水一类达标率为91.9%。累计治理水土流失328.26万亩，造林绿化 86万亩，森林覆盖率达到45%。

——人民生活持续跃升。时刻把群众冷暖放在心上，公共资源向基层延伸、向农村覆盖、向弱势群体倾斜，五年来市财政民生投入783.8亿元，比“十一五”提高2.8倍，办成一系列为民实事，其中2015年确定的十件为民实事，除个别需要深入论证外，全部完成。就业形势保持稳定，登记失业率始终控制在 4%以内。城乡居民基本养老保险制度全面建立，城镇企业退休人员基本养老金连续11年上调，城乡低保标准和城乡居民医保财政补助标准大幅提高，大病保险实现全覆盖。农村互助幸福院、城市社区居家养老服务中心覆盖率分别达到73%和75%。开工建设保障性住房6.16万套，城镇低收入住房困难家庭实现应保尽保。完成农村危房改造3.66万套，累计5.05万人实现稳定脱贫，解决了81.69万农村人口饮水安全问题。社会事业全面进步，新改扩建义务教育学校 307所，市一中迁入新址，中国环境管理干部学院实现专升本，秦皇岛职业技术学院跻身国家100所骨干职业院校。市中医院完成迁建，市精神卫生中心主体工程完工，改扩建县级医院5家、乡镇卫生院38家，新建社区卫生服务中心4个。全国公共文化服务体系示范区创建通过验收。全民健身活动蓬勃开展，成功举办马拉松等重大赛事，华夏幸福足球队冲超成功。法治秦皇岛、平安秦皇岛建设扎实推进，社会治理格局不断完善，食品药品安全和安全生产形势总体平稳，社会大局和谐稳定。圆满完成暑期服务保障工作任务，借力暑期发展实现突破。扎实开展国防动员和双拥共建工作，驻秦部队和广大民兵在森林防火、抗洪抢险等急难险重任务中作出了突出贡献。民族宗教、新闻广电出版、外事侨务、人民防空、档案、审计、统计、气象、地震、地方志、援疆、妇女儿童、老龄、残疾人等工作都取得新成绩。

——政府自身建设不断加强。坚持以新理念引领政府工作，以新思路推动发展建设，以新形象凝聚民心民力。认真开展群众路线教育实践活动、“三严三实”专题教育、解放思想大讨论，严格落实中央“八项规定”，“四风”问题得到有效整治。推动管理创新，完善科学民主决策机制，推行机关标准化管理，启动立法工作机制建设，落实政府法律顾问、重大事项专家咨询制度，加强市、县、乡、村四级政务服务体系建设，依法行政能力进一步增强。自觉接受人大法律监督和工作监督、政协民主监督和社会监督，认真解决代表委员关注的热点难点问题，累计办理国家、省和市人大代表建议、政协提案2646件，按时办复率和规范化率均达 100%。切实加强廉政建设，认真履行党风廉政建设主体责任，不断完善体制机制，加大违法违纪案件查处力度，一批腐败分子得到应有惩处。

这些成绩的取得，是省委、省政府和市委总揽全局、正确领导的结果，是全市上下团结奋斗、共同努力的结果。在此，我代表市政府向全市人民、人大代表、政协委员，向各民主党派、工商联、无党派人士和人民团体，向驻秦人民解放军、武警官兵和公安政法干警，向中省直机关驻秦各单位，向关心支持秦皇岛发展的海内外朋友们，致以崇高的敬意，表示衷心的感谢!

回顾五年来的发展历程，我们深刻体会到，只有突出发展第一要务，努力在发展中转变、在发展中调整、在发展中提升、在发展中增效，才能争得主动、有效作为，不断开辟发展新天地;只有主动认识、适应、把握、引领经济发展新常态，做到观念上适应、认识上到位、方法上对路、工作上得力，才能实现发展方式、管理形式和思维模式与时俱进;只有强化机遇意识，切实把京津冀协同发展、环渤海地区合作发展贯穿到经济社会发展的各领域各环节，才能在对接京津、服务京津中加快补齐短板，真正使机遇转化为发展优势;只有坚持创新引领，全面深化改革开放，努力破解发展瓶颈、抢占发展制高点，才能实现发展动能的加快转换，为经济社会发展注入生机活力;只有着力保障改善民生，增进人民福祉，财力向民生投放、政策向民生倾斜，切实解决人民群众最关心最现实的利益问题，使发展成果更多更公平惠及全市人民，才能为经济社会发展打牢基础;只有持续改进作风，始终牢记“两个务必”，认真践行“三严三实”，持之以恒纠“四风”，坚定不移反腐败，才能为改革发展提供坚强保障;只有坚持依法行政，在法治的轨道上开展工作，做到法定职责必须为、法无授权不可为，才能切实提高政府的公信力、执行力。

各位代表!我们必须清醒地看到，对照全面建成小康社会指标体系，当前秦皇岛经济社会发展中还存在不少短板和问题。一是发展任务十分繁重。经济总量小、发展速度慢的局面没有根本改观。特别是近年来，经济下行压力持续加大，主要经济指标增速在低位徘徊，影响了“十二五”目标的如期实现，增大了未来五年发展压力。二是发展质量亟待提高。主导产业链条短，配套能力低，创新能力不强，初级产品多、附加值不高，新兴产业占比虽有增加，还不能弥补传统产业出现的缺口，同时重大产业支撑项目不足的问题依然突出。三是环境治理任务艰巨。超标排放、偷排偷放问题时有发生，污水直排现象尚未根治，空气质量在环保部重点监测城市中尚处中下游，个别河流水质仍为劣五类，综合整治长效机制还不健全，治污任务仍旧艰巨。四是城镇化水平不高。城市规划相对滞后，滨海特色不鲜明;中心城区支撑作用不强，城市区与县城协调不够、联动不密切。五是城市精细管理尚有差距。管理体制和运行机制亟待改革，违法占地、违法建设等问题比较突出，背街小巷整治、老旧小区物业管理、交通拥堵等问题仍未得到根本解决，市民文明素质有待提高。六是社会建设存在薄弱环节。劳动就业、社会保障、收入分配、教育卫生、居民住房、公共服务等关系群众切身利益的问题仍然较多，改善困难群众生产生活条件的任务还很重。七是政府工作人员作风建设亟待加强。一些部门、干部服务意识和大局意识不强，缺乏应有的责任和担当，不作为、慢作为、不敢为、不会为。对此，我们必须牢固树立忧患意识，痛下决心，采取强力措施加以解决。二、“十三五”时期的主要任务和目标

“十三五”时期，是全面建成小康社会的决胜阶段，是建设沿海强市、美丽港城的关键时期。面对复杂多变的严峻形势，面对不进则退的竞争挑战，能否实现跨越赶超、奋进崛起，内因至为关键，理念尤其重要。必须锐意开拓进取，激发内生动力，才能战胜挑战、克难前行;必须不断解放思想、更新理念，才能开辟发展新路径、提升发展新境界。当前，京津冀协同发展、环渤海地区合作发展等重大战略的推进，为我们提供了多重叠加的机遇。国家和省对我市的关注支持，全市人民对美好生活的热切向往，为我们破解瓶颈创造了极为有利的条件。重托赋予使命，热望期待担当。我们必须以舍我其谁的责任感和时不我待的紧迫感，殚思极虑、激情工作，以一往无前的信心抢抓机遇，以战无不胜的决心迎接挑战，奋力开创改革发展新局面。

“十三五”时期，我市经济社会发展的指导思想是：全面贯彻党的十八大和十八届三中、四中、五中全会以及省委八届十二次全会和市委十一届八次全会精神，以邓小平理论、“三个代表”重要思想、科学发展观为指导，深入贯彻习近平总书记系列重要讲话精神，高举发展、团结、奋斗的旗帜，按照“四个全面”战略布局，坚持创新、协调、绿色、开放、共享的发展理念，坚守发展、生态、民生三条底线，把握协同发展、转型升级、又好又快的工作主基调，坚持稳中求进，坚持全面深化改革开放，坚持以提高发展质量和效益为中心，着力推进结构性改革，着力推进生态立市、产业强市、开放兴市、文明铸市，着力推进新型工业化、信息化、城镇化和农业现代化，着力推进全国文明城市、国家卫生城市、国家森林城市“三城同创”，坚决打赢脱贫攻坚战，确保全面建成小康社会，加快建设沿海强市、美丽港城，为谱写中华民族伟大复兴中国梦的秦皇岛篇章奠定坚实基础。

“十三五”时期，我市经济社会发展的总体思路目标，概括起来就是“五个发展、四个领先、三个高于、两个提前翻番、一个全面建成”。“五个发展”，就是牢固树立创新发展、协调发展、绿色发展、开放发展、共享发展的理念。“四个领先”，就是努力在生态环境、城乡统筹、开放水平、城市文明上走在全省最前列。 “三个高于”，就是经济保持中高速，增长速度高于全国全省平均水平，地区生产总值年均增长8%以上;发展迈入中高端，质量效益提升幅度高于周边地区;环境治理大见效，生态改善程度明显高于以往。“两个提前翻番”，就是争取到2019年生产总值比2010年翻一番以上，城乡居民人均可支配收入比2010年翻一番以上。“一个全面建成”，就是确保如期全面建成小康社会。提出地区生产总值年均增长8%以上、争取提前到2019年实现两个翻番的目标，高于全国、全省平均水平，综合考虑了全面建成小康社会的目标要求，沿海强市、美丽港城建设的客观需要以及面临的多重机遇和有利条件，是积极进取、符合实际的。

实现上述发展目标，必须适应、把握、引领经济发展新常态，牢牢抓住发展第一要务不放松，深入实施创新驱动战略，大力度推进供给侧结构性改革，在保证好的同时争取速度更快、保证质的同时争取总量更大，努力实现更高质量、更有效率、更加公平、更可持续的发展。未来五年，要着力抓好以下六大任务：

(一)坚持绿色发展，建设京津冀城市群中生态标兵城市。生态是生存之基、立市之本、发展之源。必须牢固树立“绿水青山就是金山银山”理念，坚持绿色富市、绿色富民，实施主体功能区战略，划定生态空间红线，设立产业准入负面清单，推广绿色建筑、节能建筑，协调推进全域生态化、生态产业化，创建国家生态文明先行示范区。以更强的决心推进污染防治。实施“蓝天工程”和“碧水工程”，加强联防联控和流域共治，坚决压煤、降尘、减排、节能，推进北戴河近岸海域环境和水环境深度治理。到“十三五”末，空气质量基本达到二级以上标准、进入全国先进行列;近岸海域水质、地表水达到环境功能要求，集中式饮用水源地水质达标率100%;深入开展土壤污染防治，改善土壤环境质量，高耗能、高污染、高风险的化工生产企业有序退出主城区。加强噪声污染治理，营造安静宜居环境。以更大的力度加强生态修复。抓好山水林田湖海重大生态工程，推进全域生态系统修复建设。“十三五”期间，全部关闭露天开采矿山，创建国家森林城市，森林覆盖率达到60%以上;争取青龙纳入国家重点生态功能区，祖山、都山、碣石山纳入国家公园体系，提升北戴河国家湿地公园生态功能，努力打造青龙湖等国家湿地公园和柳江世界地质公园。以更严的制度强化生态监管。建立健全生态文明制度，科学设定并严格遵守资源消耗上限、环境质量底线和生态保护红线，实行能源、水资源、建设用地的总量和强度双重控制。加强市级环境立法，强化环保行政执法和刑事司法联动，建立健全自然资源资产审计制度，实行生态环境损害责任终身追究，形成政府主导、企业明责、全民参与的生态文明建设良性循环局面。积极挖掘生态附加值，发展生态旅游、生态农业等绿色经济，把生态优势转化为发展优势。

(二)坚持转型发展，构建绿色低碳循环发展的现代产业体系。产业是城市发展的关键，是区域竞争的根本。必须突出产业这个支撑，落实“中国制造 2025”和“互联网+”行动计划，更加注重供给侧结构性改革，深度优化产业规划布局，构建先进制造业和现代服务业共同主导、传统产业提升与新兴产业发展双轮驱动的新格局。改造提升传统产业。积极化解过剩产能，加快淘汰落后技术工艺、生产设备，实施增品种、提品质、创品牌工程，推动金属压延业中高端化、装备制造业智能化、粮油食品业精细化、玻璃建材业绿色化，重振耀华品牌，重塑葡萄酒产区形象。培育壮大战略新兴产业。积极实施大数据战略，重点发展高端装备制造、信息技术、节能环保、新能源、新材料等新兴产业，培育一批新兴产业集群，到“十三五”末，战略新兴产业占规模以上工业增加值比重显著提高。加快发展现代服务业。突出旅游业的基础地位，完成旅游管理、经营体制改革，打造全市域旅游，推动“旅游+”全产业融合，山海关重塑5A景区品牌，北戴河争创国家全域旅游示范区，将我市打造成京津冀最佳旅游目的地。壮大现代物流业，建设区域性能源交易中心和农产品交易平台，推进港产城一化，打造东北亚物流枢纽;推动制造业由生产型向生产服务型转变。大力发展健康产业，建设北戴河生命健康产业创新示范区。打造足球城市，推进体育产业发展。加快发展总部经济、服务外包、研发设计、会展经济等新业态。加速农业现代化。调整优化农业结构，完善农业科技和社会化服务体系，培育新型职业农民，抓好农业基础设施和现代农业园区建设，培育家庭特色农场，实施山区综合开发工程，推动一二三产深度融合、产加销游一体发展，打造现代农业产业集群，走产出高效、产品安全、资源节约、环境友好的农业现代化道路，争创国家级现代农业示范区和生态循环农业示范市。

(三)坚持创新发展，努力实现经济发展动能的重大转换。创新是民族进步的灵魂，是引领发展的第一动力。必须把握创新这个核心，发挥科技创新在全面创新中的引领作用，激发全社会创新创业活力，推动新技术、新产业、新业态蓬勃发展，打造中国北方科技创新之城。以创新平台完善塑造发展新优势。加快聚集创新资源，积极融入京津冀协同创新，争取更多京津科技园区、研发基地落户我市，合作共建工程技术研究中心、产业研究院、技术和知识产权交易中心等平台，加快北戴河新区、海港区省级高新区建设，推动秦皇岛开发区成为京津冀创新要素聚集区。以创新主体培育强化发展支撑。支持中信戴卡、中铁山桥、哈电重装、康泰医学等优势企业增强自主创新能力，建设行业创新领军企业。依托驻秦高校和科研院所，完善产、学、研互动互促机制，实施“苗圃、雏鹰、科技小巨人、新三板上市”四大工程，培育高新技术企业和科技小巨人企业，促进科技型中小企业裂变式增长。落实“双百双千”工程，打造创新创业人才高地。大力推进大众创业、万众创新，实施“双创”三年行动计划，完善服务和政策体系，加快发展众创空间、科技企业孵化器和加速器，把秦皇岛建成北方“双创”基地，打造创客新城。以创新引领扩大有效需求。着力实施重大消费工程，优化消费环境，扩大新产品和服务供给，以供给创新释放消费潜力，以消费结构升级带动产业转型升级。着力优化投资结构，增加有效投资，提高投资效率，创新融资方式，吸引更多社会资本参与产业项目、基础设施建设，不断开辟区域发展、产业发展、网络经济、海洋经济新空间，加速培育新的经济增长点。

(四)坚持开放发展，为又好又快发展增添更加强劲的动力。更加自觉、更加坚定地推进改革开放，以开放突破发展藩篱，以改革破解瓶颈制约。切实抓住京津冀协同发展机遇。精准落实京津冀协同发展纲要，推行“1+1”模式，加强与国家部委、北京市直单位对接合作，推动优质资源与北京教育、医疗、文化、科技高端要素融合对接，加快建设“一都三区一枢纽”。精准落实环渤海地区合作发展规划，拓展陆海通道，实现与承德、张家口等区域的互联互通，打通秦皇岛港与二连浩特口岸联系，推进跨区域物流通道建设，加强金融、旅游、能源、环境整治、现代信息基础设施等方面的合作。切实提高对外开放质量和水平。严格开发区定位，沿海开发区执行“规定动作”，其他开发区实行“负面清单”。完善管理体制，积极配套基础设施，充分发挥各省级开发区的载体功能，推进“一区多园”，有效发挥开发区的龙头作用。完善招商激励政策，改革招商机制，创新招商方式，提高招商精准度，积极引进战略投资者，充分利用“两个市场、两种资源”。主动融入 “一带一路”、中蒙俄经济走廊和中韩自贸区建设，实施优进优出战略，发展服务贸易，巩固传统市场，开拓新兴市场。依托出口加工区、保税仓库等资源，争取建设综合保税区。切实加快释放改革红利。以更大决心和勇气推进各领域改革，为市场主体营造更好环境，充分发挥市场配置生产要素的决定性作用;毫不动摇地鼓励支持和引导非公有制经济发展，进一步提高民营经济增加值占生产总值的比重。

(五)坚持协调发展，构建城乡统筹、区域一体化发展新格局。城镇化是稳增长、调结构的黄金结合点。必须抓住城镇化这个重点，对标国内一流滨海城市，以 “三城同创”为动力，统筹空间、规模、产业三大结构，统筹改革、科技、文化三大动力，统筹生产、生活、生态三大布局，统筹政府、社会、市民三大主体，加快新型城镇化进程，到“十三五”末，全市常住人口城镇化率达到65%左右。高站位优化城市功能布局。以行政区划调整为契机，构建以中心城区为内核、滨海城区为两翼、县城和重点镇为支点的发展格局。理顺规划管理体制，完善城市规划体系，推进“多规合一”，引导调控城市建设和规模。高标准编制海岸线地带总体城市设计，严格控制建筑高度、体量和密度。高品质精致建设城市。全面推行城市设计，注重建筑风格和天际线的协调，从整体平面和立体空间上统筹城市建筑布局，坚决杜绝“快餐式”建筑。加强地下和地上基础设施建设，着力解决“马路拉链”等人民群众反映强烈的现实矛盾和问题。以西港搬迁改造提升为契机，推动海港、空港、路港、信息港“四港”融合，拓展国际、国内双向腹地，构建具备多式联运功能的物流枢纽，建设城市轨道交通和城乡快速交通系统，大力发展城市公共交通，完善公共综合交通体系。高水准建设新型县城。推进昌黎与北戴河新区统筹发展，推进青龙、卢龙县城产城教融合，实现县域经济增比进位，争创国家园林县城，打造京唐秦发展轴上的“微中心”。统筹乡镇建设、山区综合开发和脱贫攻坚，培育一批与景区、园区融合的特色强镇和美丽乡村，引导农村人口有序转移。高效率精细化管理城市。创新城市管理方式，完善政策法规和标准规范体系，推进城市管理大部门制改革和综合执法，提高城市治理能力和精细化管理水平，着力解决“城市病”等突出问题，确保城市管理和环卫保洁达到全国先进水平，创建国家卫生城市。开展“双违”整治三年攻坚行动，依法消灭城市建成区所有的违章建筑，坚决遏制新增违法建设。高要求培育城市人文精神。用中国梦和社会主义核心价值观凝聚共识、汇聚力量，加强思想道德建设和社会诚信建设，塑造提炼秦皇岛精神，提升市民文明素质，让讲文明、讲秩序、讲礼仪、讲诚信蔚然成风，创建国家文明城市。

(六)坚持共享发展，让人民群众过上更加幸福美好的生活。共享是实现又好又快发展的根本目的。必须加大民生投入，保障基本民生，让群众有更多获得感。实施精准扶贫脱贫工程。全面落实“六个精准”要求，举全市之力打赢脱贫攻坚战，确保2017年青龙退出贫困县行列。实施城乡居民收入提升工程。坚持居民收入和经济增长同步、劳动报酬和劳动生产率提高同步，多渠道增加居民财产性收入，着力提高中低收入群体收入水平。实施创业就业扶持工程。坚持以创业促就业，衔接落实国家、省积极就业政策，健全职业技能教育培训体系，加快构建覆盖城乡的公共就业服务体系和城乡平等的就业制度，拓展更多就业岗位。实施社会保障体系完善工程。完善城镇职工、城乡居民基本养老和基本医疗保险制度，健全城乡居民大病保险制度，实现城乡居民医疗保险市级统筹，争取实现医疗保险在京津冀区域内购药就医直接结算;健全住房保障和供应体系，提高城乡低保补助标准，大力发展社会救助、社会福利和慈善事业，确保困难群众基本生活。实施教育提质惠民工程。大力发展各级各类教育，提高教育教学质量，完善家庭经济困难学生资助体系，促进教育公平。加快产教融合发展步伐，推广“产业园区+标准厂房+职业教育”模式，兴建一批县区校企合作共建示范点。实施百姓健康共享工程。深化医药卫生体制改革，优化医疗资源布局，鼓励社会资本兴办医疗机构。着力提高医疗服务质量，积极回应群众看病难、看病贵难题，为改善医患关系觅“良方”。加快发展中医药事业。推动医养结合，积极开展应对人口老龄化行动。大力发展文化、体育事业，提升人民健康素质。实施公共安全工程。健全立体化社会治安防控体系和大维稳工作格局，推进平安秦皇岛建设。开展国家食品安全城市创建活动，确保人民群众饮食用药安全。严格落实安全生产责任，建立健全各项制度，强化隐患排查治理，加大监管执法力度，确保人民群众生命财产安全和职业健康。

各位代表!“十三五”宏伟蓝图已经绘就，决胜全面建成小康社会的号角已经吹响。我们坚信，有中央、省委省政府和市委的领导，全市人民齐心协力、奋勇拼搏，沿海强市、美丽港城建设一定能够开创新局面，全面建成小康社会目标一定能够实现!三、2016年主要工作

今年是“十三五”的开局之年，是京津冀协同发展全面推进的关键一年，也是推进结构性改革的攻坚之年。我们要认真落实中央、省委省政府和市委的决策部署，统筹把握稳增长和调结构的平衡点、结合点，在战略上坚持稳中求进、打好持久战，在战术上坚持重点突破、打好歼灭战，大力推进供给侧结构性改革，去产能、去库存、去杠杆、降成本、补短板，加快发展动能转换和质量效益提升，促进经济持续健康发展和社会和谐稳定，为实现“十三五”发展目标强势开局。

今年我市经济社会发展主要预期目标是：地区生产总值增长8%，一般公共预算收入增长7%以上，全社会固定资产投资增长12%以上，社会消费品零售总额增长8%以上，进出口总额增长5%，实际利用外资增长5%，单位生产总值能耗下降2.5%，化学需氧量、二氧化硫、氨氮、氮氧化物排放量分别削减1.5 %、2%、1%和3%，PM2.5浓度下降8%以上，城乡居民人均可支配收入分别增长8%和8.5%，城镇登记失业率和居民消费价格指数分别控制在4%和 3%以内，常住人口城镇化率达到55.6%。围绕目标的实现，重点抓好八方面工作：

(一)不遗余力稳增长，确保经济运行在合理区间。加强供给与需求有效对接，扩大有效需求，构建多点发力、多元支撑的增长动力格局，力促经济保持中高速增长。发挥投资关键作用。坚持把项目作为扩投资、调结构的重要载体，全年安排京能热电联产等重点建设项目100个、前期项目100个，60个项目开工建设、40个项目竣工投产。精准对接国家“7+4”重大工程包，制定三年滚动投资计划，建立转型发展项目储备库。制定领导带头抓项目责任办法，全面推行投资项目代办制，继续开展项目建设“三集中”活动，对重点项目实行清单式、精准化管理服务，强化调度督导、考核奖惩，确保更多项目签约落地、开工建设、投产达效。发挥消费基础作用。着力提升旅游消费，开发温泉、滑雪、山地、健康养生等多层次、多样化旅游消费产品，暑期前推出富有特色的十大旅游商品和十大旅游纪念品，打造“秦皇岛礼物”品牌。统筹旅游和商品房营销，借助开通至石家庄、邯郸、太原等地高铁契机，加大冀中南、山西等地营销力度，拓展旅游和房地产市场。深化户籍制度改革，完善促进房地产健康发展的政策措施，发展住房租赁市场，有效化解房地产库存。适应“2.5天小长假”和职工带薪休假制度，鼓励错峰休假，推动城市夜经济常态化。加快发展信息消费，推动三网融合，提高接入带宽、网络速度率和宽带普及率;发展社区电子商务、连锁经营和社区配送，实施电子商务进农村工程，发展京东商城秦皇岛特色馆等网络零售企业，推进电子商务全覆盖，电子商务交易额达到600 亿元左右。推进“互联网+快递”，加快快递业转型升级和提质增效。落实工商用电同价、税收减免等政策，增强商贸企业活力和竞争力。发挥实体经济支撑作用。落实省“百千万”助力工程，培育主营业务收入超50亿元企业6家、超百亿元企业1家。开展降本增效专项行动，制定实施降低企业成本总体方案，降低制度性交易成本、人工成本、企业税费负担、社会保险费、企业财务成本、物流成本，建设全省生产要素成本“洼地”。建立信息互动共享平台，确保各类政策信息第一时间让企业知晓、让企业受益。分业施策、精准帮扶，出台实施支持传统产业、新兴产业发展的政策措施，完善重点企业直通车服务体系，对规模以上企业重点监测服务。建立新增长点跟踪服务机制，开展中小微企业帮扶活动，健全创业辅导、人才培训和技术支持等服务平台，帮助规模以下企业提档升级，年内新增规模以上工业企业40家以上，确保规模以上工业增加值增长5%以上。深化商事制度改革，放宽市场准入，鼓励民间资本进入更多领域，激发民营经济的活力和创造力;大力优化营商环境，依法保护企业家财产权和创新收益，全面激发企业家精神。发挥园区经济带动作用。建立完善开发区动态管理考核机制，实行末位淘汰制度。淡化行政级别，推行全员招聘。加强基础设施建设，每个省级开发区都要建设投融资平台，至少设立1支产业发展基金，每年增加1—2项功能建设，两年内达到“十通一平”标准。推进园区整合，集中力量培育一批特色产业园区，每个县区开发区选定1—2个支柱产业重点发展，做大戴卡轮毂汽车零部件园区，推进美铝型材产业园区建设，加速壮大园区经济规模。强化秦皇岛开发区龙头作用，加快实施“飞地”政策，建立分享机制，推动国家级、省级开发区融合发展。

发展是硬道理，是解决一切问题的根本。我们一定积极化解“难”，努力巩固“稳”，不断推动“进”，充分挖掘和释放我市潜力，努力使“沿海强市”真正强起来!

(二)坚持不懈调结构，推动产业向中高端迈进。以增强科技创新能力为支撑，坚持“加减乘除”并举，推进产业结构调整和转型升级，释放新需求，创造新供给，培育新动能。强化科技引领作用。落实企业所得税加计扣除、企业研发费用投入后补助等科技体制改革政策措施，鼓励企业增加研发投入，支持企业自主决策先行投入开展创新活动。整合市域内企业、高校、科研机构科技资源，建立共享平台，加快发展科技型服务业。鼓励企业主导构建产业技术创新联盟，建设生物技术工程技术研究中心、健康医学产业技术研究院等平台，确保市级以上研发机构超过137家。加强与国家、省科技重大专项的对接，组织实施重点科技公关项目。鼓励支持企业“1+1”发展，围绕市场需求和供给创新，引进一项技术、新上一个项目、新建一座工厂、引进一家合作单位。实施高新技术产业倍增和科技型中小企业成长计划，引导中小微企业走“专精特新”发展道路，确保年内科技型中小企业达到1300家、高新技术企业达到100家以上。加快工业转型升级。开展制造业升级专项行动，实施100项重点技改项目，推进“两化”融合，提高装备制造、金属压延、粮油食品、玻璃建材等传统产业技术装备水平;发挥美铝渤海、中信渤铝等企业优势，打造汽车轻量化配件基地。落实扶持政策措施，全方位支持葡萄酒产业发展。实施新兴产业增比进位工程，抓好凯斯曼二期、华恒生物二期三期等项目建设，争取通用哈动力燃气轮机、福田电动客车、凯盛薄膜太阳能电池组件等项目落地，加快高端装备制造、新能源、节能环保等新兴产业发展。积极化解过剩产能，压减炼铁、炼钢、水泥产能，鼓励山桥、山船等企业利用自身优势开拓钢铁应用市场，市域内道路桥梁、市政设施和公共建筑率先使用钢结构。推进企业循环式生产、园区循环式改造、产业循环式组合。做大做强旅游产业。按照城市即景区、景区即城市的理念，高标准制定全市域旅游规划。深化旅游管理体制改革，加强风景名胜区规范管理和保护，积极推进与战略投资者的合作，完成国有旅游景区景点所有权、管理权和经营权分离。组建秦皇岛旅游开发股份公司，支持景区企业直接上市融资。完善旅游综合执法体系，成立旅游警察支队。A级以上景区全面推行质量管理体系认证，建立“红黄黑”榜制度，奖优罚劣。完善旅游通道建设，全面整治景区周边卫生、治安环境，整合智慧旅游资源，确保硬件设施、管理服务和环境卫生实现质的跃升。北戴河以争创国家全域旅游示范区为抓手，完成鸽子窝、碧螺塔公园改造提升，按景区标准实施北戴河街景整治。山海关围绕山、海、关三大元素互动，深入整改，做靓壮美长城、古韵雄关、动感海岸、福地燕山、灵秀河湖五大文化品牌，确保年内达到5A景区标准。积极推进五佛森林公园、圣蓝游艇俱乐部、海螺岛、葡萄岛、海上巴士等项目建设，与山区综合开发、发展沟域经济、建设美丽乡村相结合，整合开发碣石山、一渠百库、柳江盆地、柳河山谷等北部浅山区旅游资源，推动旅游向海、进山、入村发展。大力提升现代服务业。推进成品油、煤炭、粮食等大宗商品交易中心建设，完成水果交易中心建设，巩固秦仁航线，争取开通相关国际航线，促进区域物流基地建设，提升临港物流业发展水平。依托现有软件产业研发基础，发展软件开发与测试、运行维护、信息系统应用服务以及电信业务咨询、系统开发等服务外包，建设京津冀服务外包基地，争创国家级服务外包示范城市。加快发展动漫游戏、文化创意、休闲体育等新兴文化业态，抓好北戴河艺术村落、中俄文化艺术合作产业园、海大汽车文化创意产业园、省体育训练中心建设，推动广电传媒集团等企业健康发展。积极培育会议会展业，加快建设北戴河新区国际会议会展中心，办好第二届中国康养产业发展论坛，争取承办老龄问题国际论坛。依托燕山大学承办全省工业设计大赛，加快发展研发设计、节能环保服务等生产性服务业。推进大众创业、万众创新。加快秦皇岛开发区、北戴河区创新创业示范基地建设，每个县区建设1个人才创新创业园，完善金融、商业、生活、交通等功能配套，建设创新公寓，竭力为创新创业提供优越条件。落实我市《关于发展众创空间推进大众创新创业的实施意见》，支持北大未名、北岛博智、e谷创想空间等加快发展。设立天使投资和科技成果转化股权投资引导基金，新增创投基金2家以上。实施人力资源提升行动计划，开展创业导师秦皇岛行活动，加强公务员、企业家两支队伍建设，强化专业技术人员、大学生和农民工创新创业能力培训。

(三)抢抓机遇促协同，开创融入协同发展新局面。抢抓京津冀协同发展、环渤海地区合作发展机遇，发挥优势，补齐短板，提升能级，加快打造京津冀区域重要节点城市。推进基础设施互联互通。加快秦皇岛动车检修基地建设，开通更多至北京、石家庄等地的始发高铁，推进京秦高速二通道及北戴河新区支线等项目，进一步密切与京津、冀中南的联系。推进承秦铁路工程和秦承张通道建设，打开西北地区重要出海通道。实现北戴河机场转场通航，培育国际国内航线航班。承接非首都功能疏解。统筹北戴河区和北戴河新区功能布局，以北戴河区为核心，抢抓休疗院所改革契机，整合资源，积极承接首都经营性服务机构转移。以北戴河新区为重点，全力加快生命健康产业创新示范区、侨梦苑侨商产业聚集区建设，务实推进与北京协和、北大未名、东软集团等机构的合作，加快环科院大气污染防治研究中心建设。积极对接北京高校、医疗机构，争取北京第二外国语学院、河北医科大学等在我市设立分校。支持秦皇岛港转型升级，向物流、商贸、信息、金融等功能拓展，提升质量效益和服务水平，由单一的煤炭运输港向综合贸易港转型。积极推进西港搬迁改造提升，统筹做好东扩港区建设方案、临港物流园区控制性详细规划和西港新城区城市设计，努力引进战略投资者，推动150航道实质性开工，科学稳妥实施东扩港区建设和西港片区开发，打造京津冀滨海高端商务区和对外开放新平台。承接京津产业转移。加快推进与华润集团、首创集团合作项目建设，争取北重燃气轮机等项目落户，加快推进国家康复辅具检测中心、研究中心和国家康复医院在我市分支机构建设。发挥中关村海淀园秦皇岛分园的龙头作用，积极推动中关村自主创新示范区政策向中关村海淀园秦皇岛分园延伸，争取京津一批新的技术、资金、项目落地。谋划建设天津自贸区功能分区，强化与北京物流企业合作。系统对接、谋划推进与葫芦岛市等辽宁省际交界区域的合作，打通省际旅游通道。

(四)多措并举扶“三农”，切实提升农业农村现代化水平。加大强农惠农富农力度，持续深化农村改革，积极破解“三农”难题，开创农业农村工作新局面。提高农业综合生产能力。加强农业基础设施建设，推进高效节水灌溉工程和中低产田改造。优化种植结构，北戴河、山海关、海港区原区域所有大田玉米全部调整，北戴河新区调整50%;推进设施蔬菜提质增效，新增中药材、油料、杂粮等经济作物5万亩和高标准果品示范园1万亩。推动养殖业转型升级，抓好省级标准化示范县和标准化生产基地建设，发展生态效益型渔业，筏式扇贝养殖规模稳定在60万亩。提升农业现代化水平。加强现代农业园区建设，改造提升集发国家农业公园，编制完成县域现代农业园区发展规划，每个县区建设现代农业园区2—4个，创建市级园区1—2个，全市新增省级现代农业园区3个以上。启动实施农产品加工业倍增计划，发展粮油、果品、蔬菜、肉类等产地初加工和毛皮、水产品、中药材等深加工，推进顺鑫集团国际农产品物流园及国际食品工业园、丰科菌业等重点项目建设。大力实施良种战略，发展品牌农业，支持“三品一标”产地、产品认证，评选发布品牌农产品名录。持续深化农业农村改革。推进农村产权制度改革，年内基本完成农村土地承包经营权确权登记颁证工作;规范土地流转行为，健全完善流转服务体系，真正发挥“三县一区”产权交易服务平台作用，年内土地流转率达到28%以上。扩大农村集体村级股份制改造试点范围，逐步建立村级集体经济积累长效机制。深化供销合作社综合改革和国有林场林权制度改革。鼓励支持多种形式适度规模经营，加快培育新型经营主体，新发展农民合作社、家庭农场各100家，创建合作社市级示范社、家庭农场示范场各20家。每个行政村特别是贫困村至少建立1个股份合作经济体。推进美丽乡村建设。将所有农村列入美丽乡村建设规划，统筹推进碣石山、天马山等3个省、市级片区和棋盘山等8个县区级片区建设，继续抓好北戴河片区提升。以村庄净化、绿化、产业发展等为重点，着力推进民居改造、街道硬化、清洁能源利用等专项行动和农村生活垃圾五年整治行动，全面提升农村环境卫生、公用设施和服务设施水平。

农业农村是全面建成小康社会、实现现代化的基础。我们一定推动农业现代化与新型工业化、信息化、城镇化同步发展，厚植发展新优势，让农业农村成为大有作为的广阔天地!

(五)统筹兼顾强协调，进一步加快新型城镇化进程。坚持以人为核心，以“三城同创”为抓手，注重长远、打牢基础、重点推进，不断提升城市环境质量、人民生活质量和城市竞争力。坚持规划引领。加快推进城市总体规划修编，优化城市内部空间布局，统筹构建科学的规划体系。实施“多规合一”，以土地利用规划为基础，推进城市建设、产业、交通、生态等规划一张图管理，从源头上解决规划“打架”问题。全面实施城市设计，加强滨海岸线管控，高标准完成海岸线地带总体城市设计;坚持新老城区整体设计，老城区重在体现历史文化，新城区重在展现时代风貌。推行建筑节能新标准。按照“适用、经济、绿色、美观”的建筑方针，将文化艺术中心片区打造成全市标志性精品建筑群。切实强化规划的权威，坚决打击各类违反规划的行为。狠抓基础设施建设。实施秦抚快速路、祖山连接线、北戴河机场快速通道等工程，稳步推进城市快速路网体系建设，打造市域半小时交通圈。把秦皇岛火车站周边综合改造作为城建“一号工程”，年内确保完成公共部分建设，道路系统完备通车，启动北站房建设，彻底解决火车站周边交通混乱的问题。打通横断山路、山东堡路等一批断头路，疏通城市内循环。启动城市轨道交通项目。改造提升一批农村公路。加快北戴河西部水厂建设，完成引青三期工程，启动石河与引青济秦供水管线连接工程。海港区北部片区污水处理厂、西部污水处理厂等通水运行。统筹全市域市政配套设施建设，在北戴河新区、秦皇岛开发区，优先建设综合管廊，形成地下资源的有序管控。推进城市区重要交通节点立交改造，加强智能交通系统、公共停车场建设。科学推进慢行系统、海绵城市建设，规范城市无障碍环境建设。结合“宽带中国”“互联网+”，提高无线网络覆盖率及互联网普及率。持续加强县城和重点镇建设。加快发展县域经济，抓好一批年营业收入超20亿元的产业集群，培育一批龙头企业和区域品牌。实施县城建设三年攻坚行动，加大绿化、美化、亮化、净化力度，进一步完善县城功能、提升形象，年内所有县城全部实现集中供水、集中供热或清洁能源供热，各县污水处理厂全部达到一级A排放标准，每县创建1条特色街区、1个特色游园，推动各县争创国家级园林县城。加强重点镇建设，完善基础设施，推进满足条件的乡改镇、镇改街道办事处，鼓励有实力的企业投资、建设和运营特色小城镇，培育一批养老养生镇、会展商业镇、旅游镇、商贸镇，吸引农村人口向县城和重点镇集聚，年内全市常住人口城镇化率提高2个百分点。全面提高城市管理水平。瞄准“全国一流”目标，出台城市管理执法地方性法规，确保五一前在省内率先完成城市管理体制、综合执法体制、环卫体制、经费保障体制改革。实施“路(片)长负责制”，强化环卫保洁、市政管理、公用设施养护和城市公共空间维护，主次干道机扫率达到90%以上。狠抓秦青公路沿线、火车站周边等重点片区整治，拆除违章建筑的60%以上。新改建16个街头游园。继续开展交通秩序整治和“三车”治理，新购进公交车588辆，新开通公交线路15条，延伸20条公交线路进小区、覆盖30个小区，缓解交通拥堵问题。抓好老旧小区改造，打造50个物业管理示范小区，新建、改造30条城市支路、背街小巷，畅通微循环。

(六)扭住关键抓改革，激发市场活力和内生动力。坚持问题导向，积极推出既符合上级要求又切合我市实际的改革举措，助推供给侧结构性改革，释放改革新红利。推进行政管理改革。以“放、管、服”为重点，在原有基础上进一步取消、下放行政审批事项，做好保留审批事项监管，最大限度减少预审和前置审批，推进固定资产投资项目并联审批，全面清理规范行政审批中介服务。梳理政府职权，完善市级和部门权力清单、责任清单制度，以及动态调整机制。推广电子政务和网上办事，为企业、群众提供公平、可及的公共服务。完成公共资源交易中心建设，推进行业协会与行政机关脱钩改革。确保4月底前完成公务用车制度改革。深化财税金融改革。深化预算管理改革，实施全口径预算管理。完善市以下政府间财政收入划分体制，合理确定事权和支出责任，增强市本级调控能力。全面落实“营改增” 等税制改革，推进结构性减税和普遍性降费。设立政府投资基金，引导社会资本支持实体经济。支持鼓励各商业银行设立科技支行。加快农村信用社综合改革，支持设立村镇银行等新型金融机构，稳步推进小额贷款公司试点工作。推进秦皇岛银行增资扩股，加快“走出去”。强化财政金融风险防控，完善政府性债务管理体系，有效防范和处置重大金融风险及突发事件。建立银行服务地方经济考核奖励机制，考核结果定期在媒体公布。加强重点领域改革。逐步改变公共基础设施项目单纯依靠政府投资的方式，更好地发挥民间资本在基础设施建设、城市公共事业等领域的主力军作用，新建、在建污水处理厂全部推行PPP模式。支持符合条件的企业在多层次资本市场挂牌上市，年内上市挂牌企业达到50家。完成凯盛科技重组耀华集团工作。推进全方位对外开放。借力省承办全国工商联执委会，以及2016年中国—中东欧国家地方领导人会议、中国—拉美企业家高峰会议、唐山世界园艺博览会，包装推出一批合作项目、组织一系列小团组招商活动。加强产业链招商，开展“知名企业秦皇岛行投资考察”系列活动。创新亲商、尊商、安商举措，完善招商引资奖励办法，设立5000万元奖励资金。加大“走出去”步伐，鼓励优势企业开展境外投资和对外合作。支持康泰等企业开展跨境电子商务，支持秦冶重工、中铁山桥等企业在境外开展工程建设、承揽重大装备工程。健全口岸查验配套设施，完成电子口岸平台建设，提高口岸作业效率和服务水平。争取进口水果、肉类指定口岸资质，争取北戴河机场口岸正式开放列入国家“十三五”口岸发展规划。优化外贸结构，扩大金海粮油、正大食品等企业优势产品出口规模，抓好昌黎水产品出口质量安全示范区建设。

(七)精准发力治污染，切实打好环境治理攻坚战。坚持科学防控、精准治污，找准发展与保护的平衡点和结合点，坚定不移打好环境治理攻坚战，努力打造全国生态文明先行区。狠抓大气污染防治。实施“气化秦皇岛”行动，推进香港易高环保投资有限公司秸秆转化天然气项目，培育天然气市场替代煤炭消费。落实省道路车辆、散煤污染整治等四大专项行动，探索利用水泥窑协同处置垃圾废弃物，以港口煤堆、矿山开采、施工工地、城市及周边道路等为重点，建立扬尘污染精细化管理机制。强化移动源污染防治，鼓励发展新能源汽车，加速淘汰黄标车和老旧车辆。加强燃煤污染控制，淘汰城市建成区内35蒸吨及以下燃煤锅炉，淘汰城市周边、县城、重点镇10蒸吨及以下燃煤锅炉。继续实施农村清洁能源开发利用工程，狠抓秸秆禁烧和综合利用。推进火电、钢铁、建材等行业的脱硫脱硝与除尘综合治理。确保所有工业企业暑期前全部稳定达标，不达标的企业全部关停。建立健全环境监测预警体系，完善重污染天气应急响应机制。全年空气质量二级以上天数确保达到290天，力争达到300天。抓好水环境治理。继续推进北戴河近岸海域深度治理，实施戴河、饮马河、人造河、新河、大马坊河生态治理工程，加强污水源头治理，确保暑期前消灭污水直排现象，所有入海河流水质达到五类以上，力争全市所有浴场一类海水比例达到100%。突出造纸、淀粉、养殖等重点行业废水治理，出台城市建成区内现有污染较重企业搬迁改造或依法关闭实施方案，年底前全部取缔不符合国家产业政策、严重污染水环境的生产项目，所有工业园区全部建成污水集中处理设施并安装自动在线监控装置。划定城市蓝线管理范围，制定非法挤占河湖管理范围退出实施方案。加强饮用水源地保护，实施石河水库上游河道生态治理工程，全面取缔一、二级保护区内排污口和违规建设项目，集中式饮用水源地水质达标率达到95%以上。推进森林城市建设。创新体制机制，市财政安排奖补资金4500万元，广泛吸引社会资金投入造林绿化。坚持“见缝插绿”，点、线、面结合，实施重点片区造林、园区造林、村庄绿化、通道绿化、封山育林等重点工程，加快北戴河机场片区、海港区北部山区、沿海防护林等生态景观林带建设。年内完成造林绿化30万亩以上。加强森林防火工作。加快湿地生态保护修复与开发，全部关闭海港区北部三镇境内的露天矿山，加强城市生态和破损山体修复。严格环保监管执法。健全生态环境监测网络，完善县乡村三级网格化环境监管体系，严格落实环保工作责任，对主要企业排污情况定期公开，实行24小时在线监管，依法严厉打击环境违法行为，促进形成绿色生产方式和消费方式。

良好的生态是秦皇岛最靓丽的名片。我们一定进一步把生态文明建设融入经济建设、政治建设、文化建设、社会建设各方面和全过程，让秦皇岛青山常在、绿水长流、蓝天永驻!

(八)千方百计惠民生，全力提高人民生活质量。按照保民生、兜底线的要求，大幅压缩行政开支保民生，增进人民福祉，让人民群众得到更多的实惠。加大精准扶贫力度。确保年内5.5万贫困人口稳定脱贫，青龙达到“摘帽”标准。精准识别扶贫对象、精准推进帮扶工作，结合87个贫困村和贫困户特点，因村、因户、因人分类施策，实施产业和就业等脱贫攻坚行动。坚持一村一品，加快培育特色产业，增强“造血”功能。抓好贫困村配套建设，大力实施引水上山、引电上山、通路上山工程，为群众脱贫致富夯实基础。落实好农村低保线与扶贫线“两线合一”政策，将完全或部分丧失劳动能力的贫困人口全部纳入农村低保。广泛发动社会力量参与脱贫攻坚行动，鼓励企事业单位发挥资金、市场等优势开展帮扶，积极争取中央和省级帮扶单位给予支持。为每个贫困村都确定帮扶单位，并按家庭成立帮扶小组。完善市区对口帮扶机制，建立脱贫攻坚报告和督查问责制度，层层压实责任，级级传导压力，倒排时间，强力推进，务求实效。积极稳定和扩大就业。用好失业保险援企稳岗政策，稳定就业岗位。重点抓好高校毕业生、农村转移劳动力、退伍军人等人群就业，深入落实就业创业政策，加快秦皇岛市职技公共实训基地建设，健全社会化培训服务体系，深入实施高校毕业生就业促进计划和创业引领计划。完善创业小额担保贷款财政贴息办法，支持农民工等人员返乡创业。推动企业职工工资合理增长，落实机关事业单位工资和津补贴改革政策，大力治理拖欠农民工工资问题。充分发挥家庭服务业对促进就业的作用。年内城镇新增就业 4.82万人，农村劳动力转移就业1.7万人，城镇登记失业率控制在4%以内。完善多层次社会保障体系。实施新型农村合作医疗市级统筹，实现城镇居民医疗保险与新农合并轨。大力实施养老保险扩面提升工程，推动城乡居民与职工基本养老保险衔接工作。城镇低保标准提高到500元/月以上，农村低保标准提高到 3000元/年以上，农村五保供养标准提高到4500元/年以上。加快推进棚户区改造，将保障性住房范围拓展至乡镇教师、医护人员、乡镇政府及事业单位公职人员，分配入住“两租房”3000套，改造农村危房6000户左右。促进社会事业全面发展。着力提高教育质量，继续实施第二期学前教育三年行动计划，抓好城乡义务教育公办学校建设，推进县域初中教育向重点镇集中、高中教育向县城集中，巩固提高高中阶段教育普及水平。整合职业教育资源，启动新建一所高职院校。大力发展高等教育。加强教师队伍和师德师风建设。提高医疗服务水平，继续深化县级公立医院综合改革，推动医疗资源布局优化调整，加强社区卫生服务中心、乡镇卫生院和村卫生室建设。实施公共文化服务体系示范区建设规划，加快推进市博物馆(美术馆)建设，启动青少年活动中心、妇女儿童活动中心、科技馆、规划馆和秦皇岛大剧院建设，实施万名群文骨干培训工程、政府买单百姓看戏工程，打造一批文化艺术精品。深入推进全民健身工程，完成100个行政村体育器材安装工作。做好华夏幸福足球队主场服务保障工作。全面提高产品、工程、服务、环境质量，创建全国质量强市示范城市。维护社会和谐稳定。健全社会风险防控体系，提高动态监测和实时预警能力，着力防控各类风险。推进国家食品安全城市创建工作，加强食品药品安全基层网格化监管，大力开展食品药品安全专项整治，严厉打击制假售假等违法行为。强化属地、行业和主体责任落实，强力推进智慧安监暨隐患排查治理、安全诚信体系建设和安全标准化创建，深化高危行业和重点领域专项整治，深入推进职业安全健康监督管理，加大“打非治违”力度，确保不发生重大以上安全生产事故。加强法治宣传教育，提高公民法律素质。建立多元化矛盾纠纷解决机制，做好排查调处工作。完善社会治安防控体系，推进智慧网格建设，依法严厉打击各类违法犯罪活动。高标准完成暑期服务保障任务，做好借力暑期工作。加强国防教育和国防后备力量建设，全力支持部队深化改革，深入开展双拥共建活动，推进军民融合发展。支持工会、共青团、妇联、科协等人民团体发挥桥梁纽带作用，做好新闻出版、广播电视、老龄、残疾人、妇女儿童、气象、防震减灾、民族宗教、外事侨务、人民防空、邮政通信、审计、统计、地方志、档案和援疆等工作，推进各项事业取得新进步。

保障和改善民生没有终点站，只有连续不断的新起点。我们一定以更大力度、更实举措，织密织牢基本民生保障网，全力办好十件为民实事，努力让全市人民过上更加幸福美好的生活!四、加强政府自身建设

适应新常态、落实新定位、迈向新目标，各级政府使命光荣，责任重大。我们一定以改革创新的开拓意识，昂扬向上的精神状态，朝夕勤勉、永不懈怠，不断开创政府各项工作的新局面。

(一)解放思想，敢创新、求突破。坚持把学习贯彻习近平总书记系列重要讲话精神作为首要任务，激活解放思想、科学发展的“源动力”。牢固树立创新、协调、绿色、开放、共享的发展理念，严格对照“八破八立”，坚持问题导向，积极探索新常态下做好经济工作的方法和路径，把握好工作的“时、度、效”，努力在优化结构、增强动力、化解矛盾、补齐短板上取得新突破。

(二)转变职能，强管理、优服务。以敬民之心行简政之道，着力破除审批“当关”、公章“旅行”、公文“长征”等乱象，坚决取消一切束缚发展的行政权力，砍掉各类无谓证明和繁琐手续，能放的行政权力全部下放，所有的行政服务全部下沉。搞好事中事后监管，加强政务服务体系建设，深化机关标准化建设，大力推进电子政务和政务公开，使政府服务更透明、更便捷。

(三)依法行政，重法治、严规范。加快推行权力清单、责任清单和负面清单制度，坚决纠正不作为、乱作为，坚决克服懒政、怠政，坚决惩处失职、渎职。认真接受人大的法律监督、工作监督和政协的民主监督，虚心接受社会监督和舆论监督。全面落实政府法律顾问、专家咨询论证、重大事项公示和听证制度，进一步健全政府依法决策机制。深化行政执法体制改革，强化执法监督。加强行政复议工作，推进行政机关负责人出庭应诉制度。

(四)改进作风，敢担当、有作为。坚持把作风建设抓到底，一寸不让落实中央“八项规定”，坚决反对“四风”。坚决治理庸政怠政懒政问题，向不作为、慢作为、不敢为、不会为现象“亮剑”，为敢于担当者担当，让敢于担当者“无牵挂”“不吃亏”。认真践行“三严三实”，把夜以继日、担当有为作为常态，卯足劲头抓落实，消除“中梗阻”，打通制约发展“最后一公里”。

(五)廉洁清正，守底线、自身硬。严格遵守政治纪律和政治规矩，全面落实党风廉政建设主体责任和监督责任，健全廉政风险防控体系。坚持从源头入手，紧紧盯住行政审批、工程项目、惠民资金等重要领域和关键环节，管好权、管好钱、管好人。坚持有腐必惩、有贪必肃，持续保持惩治腐败的高压态势，解决群众身边的腐败问题，做到发现一起查处一起，同时标本兼治，着力立“明规”、破“潜规”，努力弘扬清风正气，营造良好的政治生态。

各位代表!今后五年是秦皇岛全面建成小康社会的关键时期，美好的愿景鼓舞着我们，神圣的使命感召着我们，人民的期待激励着我们。让我们更加紧密地团结在以习近平同志为总书记的党中央周围，在省委省政府和市委的领导下，团结依靠全市人民，开拓创新、真抓实干，攻坚克难、善作善成，为实现全面建成小康社会目标，为建设沿海强市、美丽港城而努力奋斗!
